# Interferon-γ exposure of human iPSC-derived neurons alters major histocompatibility complex I and synapsin protein expression

**DOI:** 10.3389/fpsyt.2022.836217

**Published:** 2022-09-14

**Authors:** Adam Pavlinek, Rugile Matuleviciute, Laura Sichlinger, Lucia Dutan Polit, Nikolaos Armeniakos, Anthony Christopher Vernon, Deepak Prakash Srivastava

**Affiliations:** ^1^Department of Basic and Clinical Neuroscience, Institute of Psychiatry, Psychology and Neuroscience, King's College London, London, United Kingdom; ^2^MRC Centre for Neurodevelopmental Disorders, King's College London, London, United Kingdom

**Keywords:** interferon-γ, MHCI, synapsin, iPSC, maternal immune activation, inflammation, C4A, schizophrenia

## Abstract

Human epidemiological data links maternal immune activation (MIA) during gestation with increased risk for psychiatric disorders with a putative neurodevelopmental origin, including schizophrenia and autism. Animal models of MIA provide evidence for this association and suggest that inflammatory cytokines represent one critical link between maternal infection and any potential impact on offspring brain and behavior development. However, to what extent specific cytokines are necessary and sufficient for these effects remains unclear. It is also unclear how specific cytokines may impact the development of specific cell types. Using a human cellular model, we recently demonstrated that acute exposure to interferon-γ (IFNγ) recapitulates molecular and cellular phenotypes associated with neurodevelopmental disorders. Here, we extend this work to test whether IFNγ can impact the development of immature glutamatergic neurons using an induced neuronal cellular system. We find that acute exposure to IFNγ activates a signal transducer and activator of transcription 1 (STAT1)-pathway in immature neurons, and results in significantly increased major histocompatibility complex I (MHCI) expression at the mRNA and protein level. Furthermore, acute IFNγ exposure decreased synapsin I/II protein in neurons but did not affect the expression of synaptic genes. Interestingly, complement component 4A (*C4A*) gene expression was significantly increased following acute IFNγ exposure. This study builds on our previous work by showing that IFNγ-mediated disruption of relevant synaptic proteins can occur at early stages of neuronal development, potentially contributing to neurodevelopmental disorder phenotypes.

## Introduction

Human epidemiological studies and animal models suggest a link between maternal immune activation (MIA) and an increased risk for psychiatric disorders with a putative neurodevelopmental origin, including schizophrenia and autism (1). Although there are many plausible factors that are critical for establishing neurodevelopmental resilience or susceptibility to MIA (2), there is evidence to suggest that the intensity of the maternal immune response is one important factor linking maternal infection to the potential for differential brain development and behavioral phenotypes (3–5). Indeed, animal MIA models display deficits in cognitive and social behaviors (6), which are accompanied by altered synaptic plasticity, decreased synaptic protein levels, and reduced dendritic spine density, predominantly in the prefrontal cortex and hippocampus (7–11). These findings are consistent with *in vivo* neuroimaging evidence for reduced synaptic density, as measured by reduced binding of positron emission tomography (PET) radioligands targeting synaptic vesicle glycoprotein 2A (SV2A) in schizophrenia (12, 13), reduced dendritic spines (14), and a meta-analysis confirming decreased expression of synaptic proteins in *post-mortem* brain tissue from individuals with schizophrenia (15).

One key feature of the maternal immune response that shapes these phenotypes is the elevation of numerous cytokines in the maternal serum, placenta and fetal brain (16, 17). Consistent with this view, elevated levels of cytokines in the maternal serum are predictive of the risk for the affected offspring to develop schizophrenia (18). The emerging theme from such studies is that changes in maternal cytokines during pregnancy can have long-lasting consequences (19–21). However, to what extent specific cytokines are necessary and sufficient for these effects remains unclear. Moreover, the underlying molecular mechanisms the are exerted on the developing brain and on specific cell types, remain to be fully elucidated. Evidence from animal models of MIA provides support for the involvement of altered levels of interleukins, particularly interleukin-(IL)-6, IL-1beta and IL-10 but also for the cytokines TNF-alpha, and interferon-γ (IFNγ) (6, 22–24). Of these, IFNγ has been found to have increased levels in the plasma of first-episode schizophrenia patients (25). In addition to its key role in the response to viral infection, IFNγ has also been shown to induce retraction of dendrites and inhibit synapse formation in the central nervous system (26, 27). Despite these findings, it is unclear whether and how elevated levels of IFNγ impact the development of neurons, and if this could contribute to increased risk for schizophrenia.

We previously demonstrated that acute exposure of neural progenitor cells (NPCs) and neurons derived from human induced pluripotent stem cells (iPSCs) to IFNγ results in gene expression changes in genes associated with schizophrenia and autism, and altered neuronal morphology in exposed neurons (28). In particular, IFNγ treatment increased major histocompatibility complex I (MHCI) expression (28). Class I MHC family molecules are best known for their function in presenting antigens to T-cells (29). MHCI is however also expressed in neurons and neural progenitors and has been found to be important in neuronal plasticity and for the co-regulation of synapse pruning in mice (29, 30). Furthermore, MHCI negatively regulates synapse density in developing cortical neurons, with *in vitro* manipulations of MHCI expression inversely affecting the density of both GABAergic and glutamatergic synapses in rat and mouse cultures (31). In a mouse model of MIA, synapse number in cultured cortical neurons were decreased, and MHCI was found to be required for this MIA-induced effect on synapse density (32). Genome-wide association studies (GWAS) also demonstrate that genetic variation within the MHC loci links with schizophrenia risk (33, 34). For example, variation of complement component 4A (C4) at the MHCIII locus and human leukocyte antigen-B (HLA-B) at the MHCI locus is strongly associated with increased risk for schizophrenia (35).

In our previous work, gene expression changes following IFNγ treatment included increased expression of MHCI genes and downregulation of genes related to the gene ontology (GO) term “synapses” in exposed iPSC-neurons (28). Given that IFNγ has been shown to affect expression of synaptic genes in iPSC-neurons in the absence of glial cells, we aimed to further characterize the effect of IFNγ treatment in developing human glutamatergic neurons, and specifically on MHCI and synaptic protein expression. Using Neurogenin 2 (NGN2) optimized inducible overexpression ioGlutamatergic iNeurons (*NGN2*-iNs) (36), we find that acute exposure to IFNγ activates a STAT1-signaling pathway in immature *NGN2*-iNs. Furthermore, we observed that IFNγ exposure increased MHCI protein and *HLA-B* and *C4A* expression but decreased the expression of the synaptic proteins synapsin I and synapsin II in cell bodies without altering the expression of a select panel of synaptic genes. These data further demonstrate that elevated levels of IFNγ are capable of disrupting the expression of synaptic proteins and impacting the development of immature glutamatergic neurons in the absence of glial cells.

## Methods

### Human iPSC culture, neuralization, and treatment

The ioGlutamatergic male neurotypical stem cell line (36) was obtained from BitBio (Cambridge, UK) under MTA agreement. ioGlutamatergic cells were maintained in Stemflex media (Gibco; A3349401) on six-well plates coated with 1:100 Geltrex (Life technologies; A1413302). Media was changed every 48 h and passaged when 70–80% confluent with HBSS and Versene (Gibco; 15040066) at 37°C before being transferred into new Stemflex medium. Neuralization was conducted based on the protocol used by Pawlowski et al. (36). Cells for experiments were terminally plated onto 6-well-plates (for RNA and protein extraction) or glass coverslips in 24-well-plates (for immunocytochemistry) coated with Poly-D-Lysine (5 μg/ml, PDL, A-003-E; Millipore) and laminin (1 mg/ml Sigma L2020). Human iPSCs were dissociated with accutase (A11105-01; Thermo Fisher Scientific) before being diluted with medium and subsequently resuspended in N2 medium with 1 μg/ml doxycycline hyclate and 10 μM ROCK inhibitor (Sigma; Y0503). Cells were plated at a density of 900,000 cells/well for RNA extraction and 25,000 cells/well for ICC. The cells were incubated at 37°C; 5% CO2; 20% O2 with daily N2 media changes supplemented with 1 μg/ml doxycycline hyclate. Either 25 ng/ml IFNγ (Abcam, AB9659; diluted in DMEM) for treatment conditions or vehicle (DMEM) was added at day 3 to the N2 medium. The cells were incubated for 24-h before sample collection (28). For western-blotting, total protein was extracted 15 min after treatment with IFNγ or vehicle on day 3.

In parallel, the 127_CTM_01 human iPSC male neurotypical line (37) was differentiated into NPCs using a dual SMAD inhibition protocol (37, 38). Briefly, the NPCs were expanded from day 18 frozen stocks in maintenance medium (1:1 N2:B27, 10ng/ml bFGF) for seven days. Before treatments, the cells were plated on 12-well NUNC^TM^ tissue culture plates (Thermo Scientific; 150628) at a density of 500,000 cells/well, with dedicated wells for treatment and vehicle treatments. The day after plating, the cells were exposed to 25 ng/ml IFNγ or vehicle and incubated for 24-h before sample collection.

### Western blotting

Cell lysates from treated *NGN2*-iNs were prepared from day 3 cells following treatment. Cells were lysed in RIPA buffer (150 mM NaCl, 10 mM Tris-HCl (pH 7.2), 5 mM EDTA, 0.1% SDS (weight/volume), 1% Triton X-100 (volume/volume), 1% deoxycholate (weight/volume), and inhibitors), before being sonicated with 10 short bursts. Sample buffer was added to all samples, which were then denatured for 5 min at 95°C and stored at −80°C until used further. All samples (5 μg) were subsequently separated by SDS-PAGE and analyzed by Western Blotting with antibodies against phospho-STAT1, phosphor-ERK1/2, ERK1/2, and GAPDH (Supplementary Table 1). Western blots were visualized using Clarity Western ECL substrate (Bio-Rad) before protein detection using the ChemiDoc XRS+ imaging system using ImageLab™ software. Quantification of bands was performed by measuring the integrated intensity of each band and normalizing to the housekeeper GAPDH using ImageStudioLite.

### Immunocytochemistry

Cells were fixed with 4% formaldehyde in PBS-sucrose for 10 min at room temperature, washed 2× with Dulbecco's PBS (DPBS, Gibco), and then fixed with ice cold Methanol at 4°C for 10 min, then washed 2× with DPBS. Cells were permeabilized and blocked using 2% normalized goat serum (NGS) in DPBS with 0.1% triton x-100 for 2 h. Antibody solutions (Supplementary Table 2) were prepared in 2% NGS in DPBS. The coverslips were incubated with primary antibody solution at 4°C overnight, then washed 3× with DPBS for 10 min each and incubated with secondary antibodies for 1 h at room temperature. The coverslips were washed 3× with DPBS for 10 min each and incubated for 5 min in DAPI solution, followed by two DPBS washes, then mounted onto glass slides using ProLong Gold antifade reagent (Invitrogen P36930).

### Microscopy and image analysis

Coverslips were imaged using a Leica SP5 confocal microscope. The gain and other imaging parameters were set using the vehicle control and were not changed during subsequent imaging of the control and IFNγ exposed coverslips with 246.5x246.5 μm regions imaged. The Z-stack thickness was kept at 0.5 μm and Z-stacks were then maximally projected to form a single image in FIJI. Prior to measuring fluorescent intensity, the background of each image and channel was measured in FIJI by selection of 10 25×25-pixel areas of background and measuring the mean and standard deviation (SD) of staining intensity of each area. The mean of these measurements + 2SD was then subtracted from the image. Cell Profiler (39) was used to identify the nuclei, cells, cell bodies, processes, and the cytoplasm and to measure the mean intensity of the MHC and synapsin I/II channels. Mean intensity values of 0 were excluded from the analysis. The pipeline is provided as a Supplementary file.

### Quantitative PCR

Cells for RNA extraction were lysed in TRI Reagent (T3809, Merck) for 5 min at room temperature and RNA was extracted from TRI Reagent according to the manufacturer's protocol. Isolated RNA was cleaned by precipitation with 3% sodium acetate in ethanol at −80°C overnight, washed as in the isolation protocol, and resuspended in H_2_O. A nanodrop spectrophotometer was used to measure RNA concentration and quality.

For cDNA synthesis, a mixture of 1 μl of oligo(dT)20 (50 μM) (Invitrogen; 18418020), 2 μg total RNA, 1 μl 10 mM dNTP Mix (10 mM each) (Invitrogen; 18427013), and water to make up a total of 13 μl per sample was heated to 65°C for 5 min and incubated on ice for 1 min. Next, superscript mastermix (Invitrogen; 18080093) was added to each sample (4μl 5X First-Strand Buffer, 1 μl 0.1 M DTT, 1 μl RNaseOUT Recombinant RNase Inhibitor (Invitrogen; 10777019), 1 μl of SuperScript III RT (200 units/μl)) and the mixture was incubated at 50°C for 50 min and then 70°C for 15 min. qPCR was done in a 348 well-plate, with two technical replicates per sample, and also a blank well-containing no cDNA for each primer pair (Supplementary Table 3). Three housekeeping genes (HPRT, SDHA, RPL27) were used. A mastermix consisting of 2 μl 5x qPCR Mix Plus, 1.5 μl Primer mix, and 4.5 μl RNAse free per well was added to the plate. 2 μl cDNA were added to each well. qPCR was run using a QuantStudio7 thermocycler with one cycle for 12 min at 95°C and 40 cycles of 95°C for 15s, 60–65°C for 20s and 72°C for 20 s.

The data were analyzed using the 2^−ΔΔCt^ method (40). For each gene, the technical replicates were averaged. The three housekeeping genes were averaged and the ΔCt (difference between the housekeeper average and gene of interest average) was calculated for each gene of interest. The ΔΔCt was calculated as ΔCt-[Calibrator] where the calibrator is the average of the ΔCt of the controls. The final result is 2^−ΔΔCt^. This value was log-transformed prior to statistical analysis.

### Statistical analysis

For both the ICC and qPCR experiments, three biological replicates (*N* = 3) were analyzed, where each replicate is the same cell line but with a different passage number and differentiated on a different day. The number of replicates was decided prior to the conducting of the experiments. Statistical analysis was done in Prism 9.0.2. The exposed and control mean intensity values (ICC) or log (2^−ΔΔCt^) values (qPCR) were compared using multiple 2-tailed unpaired *t*-tests, and corrected for multiple comparisons using the Holm-Šídák method.

## Results

### Acute IFNγ exposure downregulates presynaptic genes associated with synaptic vesicles

**Figure 1:**
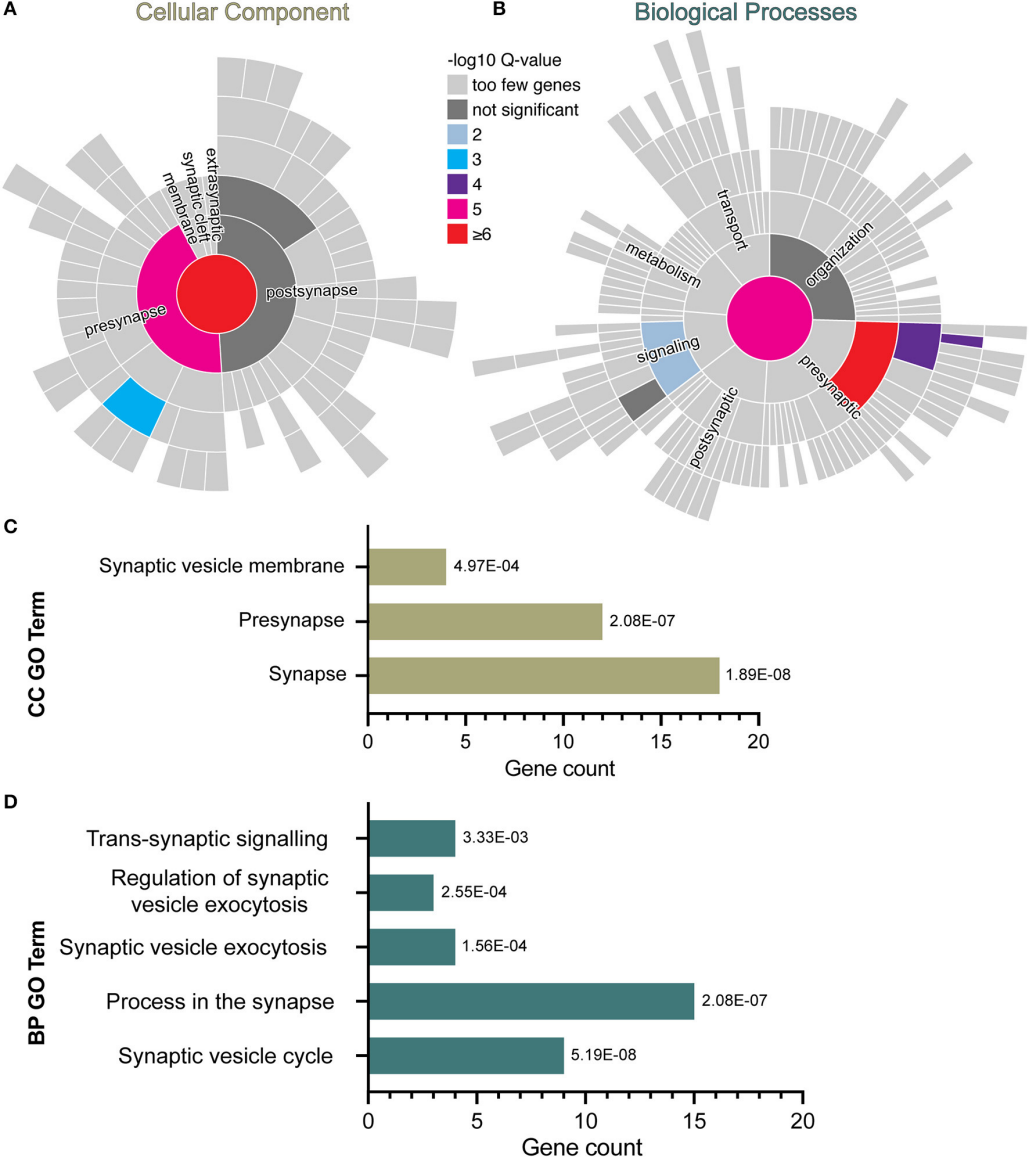
SynGO synaptic gene ontology analyses of down-regulated DEG in D30 neurons acutely exposed to IFNγ. The sunburst plots represent synaptic annotated ontologies for CC **(B)** and BP **(A)** terms. The key color scale indicates -log10 FDR adjusted *p*-values. **(A)** Significantly enriched CC ontologies include synapse (red) presynaptic (magenta) clusters. **(B)** Annotated BP terms include synaptic (magenta), presynaptic (red) and signaling (blue) terms. **(C,D)** Plots of synaptic GO output showing the 3 CC **(C)** and 5 BC **(D)** significantly (FDR-adjusted) enriched terms for D30 IFNγ exposed neurons. The bar length indicates the number of genes, the order of each bar and numbers adjacent to each are the FDR adj. *P*-value. Analysis of data previously published inWarre-Cornish et al. (28).

In the RNA sequencing data from our previous study, we found downregulation of genes related to the GO term “synapses” in human iPSC-NPCs exposed to IFNγ for 24 h (28). To explore this further, a curated database of synaptic genes, SynGO (41), was used to identify significantly enriched biological processes (BP) and cellular component (CC) ontologies related to synaptic function. Analyses were carried out with the complete list of significantly down-regulated genes in day 30 neurons acutely exposed to IFNγ (25 ng/ml, 24 h) compared with vehicle-exposed neurons. The results reveal 18 genes mapping to SynGO synaptic proteins with significant enrichment for 3 CC and 5 BP terms (Figure 1). Most of these proteins (*n* = 12) were annotated in the presynapse cluster with four genes enriched for the synaptic vesicle membrane term. These results suggest that acute IFNγ exposure leads to the downregulation of 18 genes that exert presynaptic functions and regulate synaptic vesicle mechanisms in iPSC-neurons.

### *NGN2* overexpression generates early glutamatergic neurons at day 4

**Figure 2:**
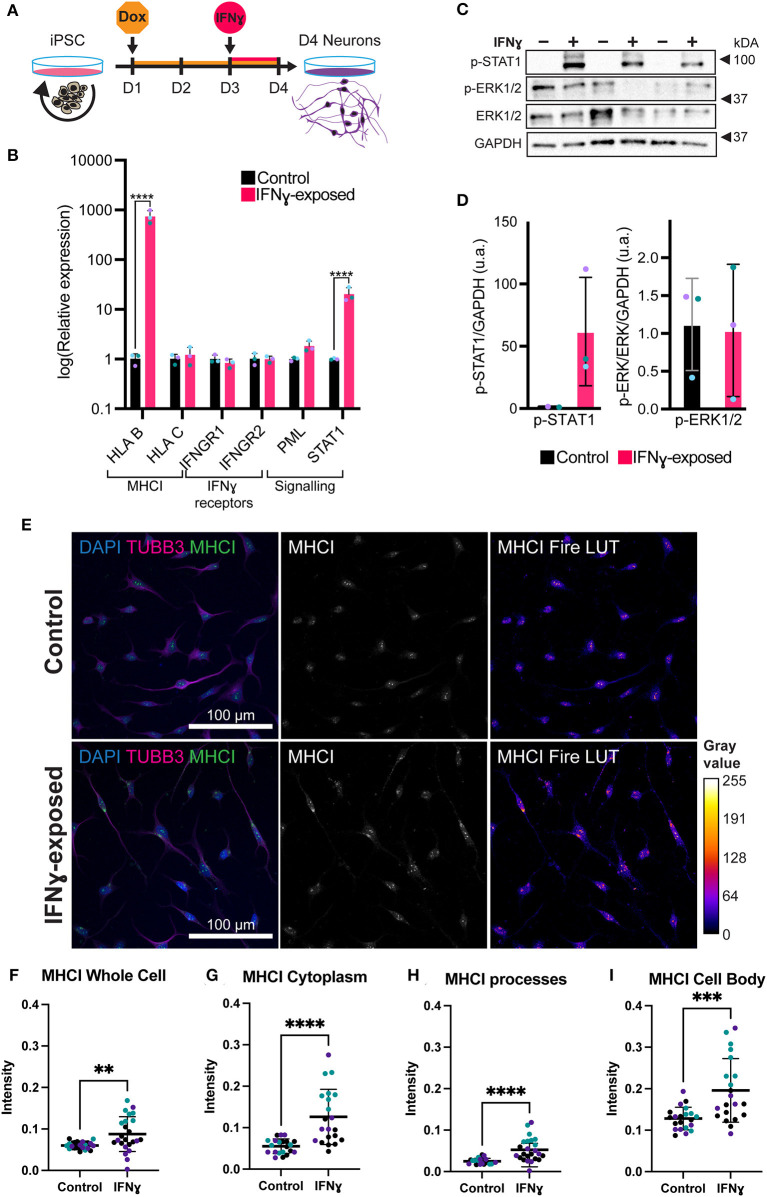
Exposure of neurons to IFNγ results in increased MHCI. **(A)** Schematic of Opti-OX neural induction and IFNγ exposure at day 3 for 24 h. **(B)** Bar graphs of relative expression of selected IFNγ signaling-related genes, showing increased *HLA-B* and *C4A* expression in IFNγ-exposed neurons. The bars indicate the log(2^−ΔΔCt^), which indicates the expression relative to housekeepers and normalized to the housekeepers of the control samples (See methods for details.) Expression in Day 4 *NGN2*-iNs exposed at day 3. *N* = 3. ^****^ indicates *P* < 0.0001, ^***^ indicates *P* = 0.000291 (unpaired *t*-test). **(C)** Western blot for p-STAT1, p-ERK1/2, ERK1/2, and GAPDH protein in Day 3 *NGN2*-iNs exposed to IFNγ (+) or vehicle (-) at day 3 for 15 min. Three biological replicates with different passage numbers are shown **(D)** Quantification of p-STAT1 and p-ERK1/2 blots shown in **(C)**. The different data point colors represent biological replicates with different passage numbers. **(E)** ICC for MHCI. The top row shows control cells, the bottom row shows cells exposed to IFNγ at day 3 for 24hrs. The MHCI Fire LUT pseudo color shows higher intensity with warmer colors and lower intensity with cooler colors. The gray values corresponding to the colors are shown on the calibration bar on the right. **(F–I)** Scatter plots of MHCI intensity in control and IFNγ-exposed neurons. The horizontal bars represent the mean, the error bars represent the standard deviation. Each point in the intensity plots represents the mean intensity of one field of view i.e., image, of the respective object. The different data point colors represent biological replicates with different passage numbers. The IFNγ and control were compared using an unpaired *T*-test, where *N* = 3 and ****indicates *P* < 0.0001, ***indicates 0.001 <*P*>0.001, **indicates 0.001 <*P*>0.01.

We used ioGlutamatergic line cells with *NGN2* optimized inducible overexpression to allow for rapid and reliable generation of *NGN2*-induced neurons (*NGN2*-iNs) upon treatment with doxycycline (Figure 2A) (36, 42). We first validated whether the ioGlutamatergic line expresses relevant markers of glutamatergic neurons after the activation of the *NGN2* gene. By day 7 of differentiation, the cells express the pan-neuronal marker microtubule-associated protein 2 (MAP2) and excitatory presynaptic marker vesicular glutamate transporter 1 (VGLUT1) (Supplementary Figure 1). After 28 days of differentiation >99% of DAPI+ cells were immune-positive for MAP2 and also expressed TBR1, VGLUT1, CAMKIIA, and SV2A, consistent with the generation of forebrain glutamatergic neurons (Supplementary Figure 2). This is consistent with evidence that the majority of mature ioGlutamatergic neurons represent cortical excitatory neurons (42, 43). Analysis was conducted on cells at day 4 of differentiation, hereafter referred to as Day 4 *NGN2*-iNs. At this developmental timepoint, the *NGN2*-iNs resemble NPCs or early neurons with synapse growth cones (43), suitable for analysis of synaptic vesicles and synapse development.

We characterized Day 4 *NGN2*-iNs using immunocytochemistry (ICC) and quantitative PCR (qPCR). We stained for the post-mitotic neuron marker neuronal nuclei antigen (NeuN) and mature neuron marker microtubule-associated protein 2 (MAP2) and the neuroprogenitor markers nestin (NES) and PAX6. In addition, staining was conducted for the early neuron/late progenitor marker Class III β-Tubulin (TUBB3). Qualitatively, all imaged Day 4 *NGN2*-iNs expressed both the neuroprogenitor markers nestin and PAX6 and the neuronal markers NeuN and MAP2 (Supplementary Figure 3), indicating that the Day 4 *NGN2*-iNs represent early post-mitotic neurons. Morphologically, the Day 4 cells had extensive processes, and some resembled young neurons with a pyramidal cell body. Other cells had a bipolar neuroprogenitor-like morphology (Supplementary Figure 3).

qPCR for the neuronal markers *NeuN, TBR1*, and *MAP2* and the neuroprogenitor markers nestin and *PAX6* (Supplementary Figure 3) shows that neural genes had a higher expression level compared to the progenitor genes, in particular *TBR1* and *NeuN* were highly expressed. Overall, these results indicate that Day 4 *NGN2*-iNs resemble early neurons.

### Acute IFNγ signals through a canonical signaling pathway in NGN2-iNs

In neurons, IFNγ is thought to signal via a signal transducer and activator of transcription 1 (STAT1)-dependent pathway, which in turn regulates the transcription of target genes (44). We thus tested whether IFNγ signaled through this canonical pathway in *NGN2*-iNs (Figures 2C,D). First, we assessed phosphorylated STAT1 levels following 15 min of IFNγ exposure. As expected, we observed increased phosphorylation of STAT1 in *NGN2*-iNs after 15 min of IFNγ-exposure (Figures 2C,D). No increased phosphorylation of extracellular signal-regulated protein kinase 1/2 (ERK1/2) was observed after 15 min of IFNγ exposure. Consistent with our previous work (28), we further observed an increase in *STAT1* and *HLA-B* expression after 24 h of treatment with IFNγ (Figure 2B). IFNγ treatment has no effect on the expression levels of the IFNγ receptors *IFNGR1* and *IFNGR2*; a trend toward increased expression of PML was also observed (Figure 2B). We further measured the expression of downstream target genes that show a robust response to IFNγ, *HLA-B* and *HLA-C*, using qPCR. Of these, *HLA-B* (t_(4)_= 27.97, *P* = 0.00001) was significantly increased in the exposed neurons (Figure 2B). Together, these data indicate that IFNγ is capable of signaling via the canonical STAT1-dependent signaling pathway in *NGN2*-iNs.

### Acute IFNγ-treatment increased MHCI but decreased synapsin I/II expression in *NGN2*-iNs

We next examined the distribution of MHCI in *NGN2*-iNs following treatment with IFNγ for 24 h. Under baseline conditions, MHCI localized to the cell body, processes, and growth cones of all Day 4 *NGN2*-iNs (Figure 2E). Consistent with our previous work (28), IFNγ-exposure caused a higher expression of MHCI in Day 4 *NGN2*-iNs compared to the control (Figure 2E). There appeared to be increased expression of MHCI in the cell body and increased MHCI localization to the processes in the IFNγ-exposed neurons. Analysis of MHCI in different sub-cellular compartments revealed that mean MHCI expression was increased by 31.2% in the cells as a whole (t_(43)_= 2.920, *P* < 0.0001); increased in the cytoplasm by 56.3% (t_(40)_= 4.723, *P* < 0.0001); cell body by 34.6% (t_(40)_= 3.819, *P* = 0.0005); and in neurite processes by 52.5% (t_(40)_= 4.331, *P* < 0.0001) (Figures 2F–I). MHC I intensity in the nucleus was not significantly different (t_(43)_= 1.937, *P* = 0.06). No change in neurite morphology was observed 24 h after IFNγ exposure (Supplementary Figure 4).

**Figure 3:**
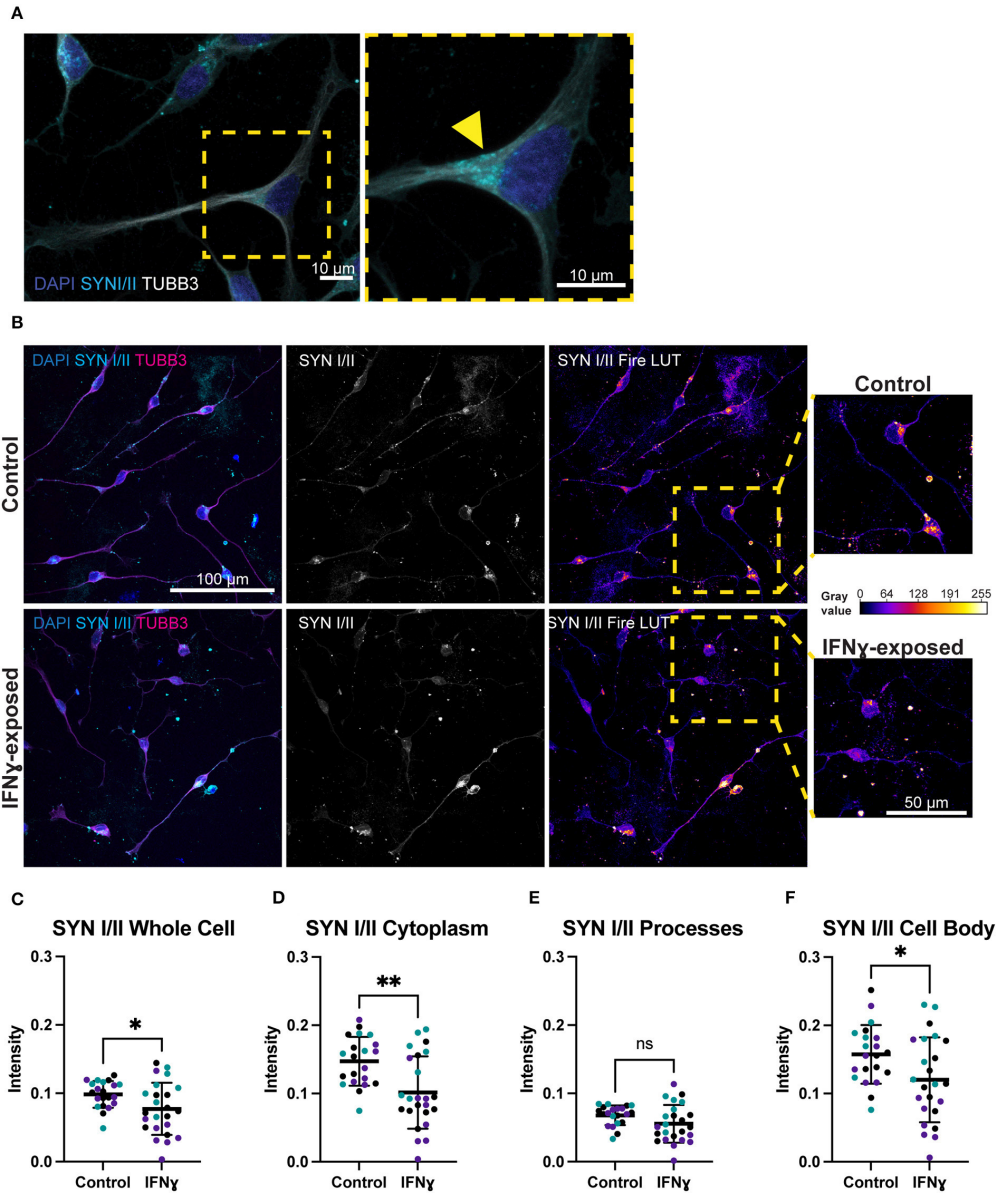
Exposure of neurons to IFNγ results in decreased SYNI/II intensity in the cell bodies of some cells. **(A)** Characteristic localization of synapsin I/II in the cell body. Right image shows a detailed view of the highlighted region. The arrowhead indicates apparent synapsin vesicles within the cytoplasm. **(B)** IHC for synapsin I/II. The top row shows control cells, the bottom row shows cells exposed to IFNγ at day 3 for 24hrs. The SYN1 Fire LUT pseudo color shows higher intensity with warmer colors and lower intensity with cooler colors. Detailed view shown on right. The gray values corresponding to the colors are shown on the calibration bar on the right. **(C–F)** Scatter plots of synapsin I/II intensity in control and IFNγ-exposed neurons. The horizontal bars represent the mean, the error bars represent the standard deviation. Each point in the intensity plots represents the mean intensity of one field of view i.e., image, of the respective object. The different data point colors represent biological replicates with different passage numbers. The IFNγ and control were compared using an unpaired *t*-test, where *N* = 3 and **indicates d0.001 <*P*>0.01, *indicates 0.01 <*P*>0.05, and ns indicates *P* ≥ 0.05 (not significant).

Given the effects of IFNγ on synaptic genes and particularly on synaptic vesicle mechanisms, we next directly tested the effect of acute IFNγ exposure on the synaptic vesicle regulators synapsin I and II, in Day 4 *NGN2*-iNs. Synapsin was selected as an early synaptic marker, since this protein is expressed in NPCs and colocalizes with constitutively recycling vesicles along the whole surface of developing axons that then localize to forming synapses (45, 46). In day 4 *NGN2*-iNs, synapsin I/II staining was localized to the cell body, processes, and growth cones (Figure 3A). Staining was particularly evident in the cell body, with synapsin I/II asymmetrically localized within the shaft of one process in many neurons (Figure 3A), presumably in vesicles being transported to the processes (Figure 3A, arrowhead). Synapsin I/II was primarily localized to the cytoplasm of the cell body. There were also sparse puncta of synapsin I/II within cell processes. Expression of synapsin at day 4 is thus primarily in the cell body of all cells.

In contrast to the effects on MHCI, synapsin I/II appeared to be decreased in IFNγ-exposed neurons. Specifically, the asymmetrically localized clusters of synapsin I/II vesicles in the shaft and cell body appeared reduced in some exposed neurons, while others had intensity that is similar to control neurons (Figure 3B). Quantification showed that synapsin I/II expression was decreased in the whole cell by 21.6% (t_(43)_= 2.303, *P* = 0.0261), cell body by 23.7% (t_(43)_= 2.300, *P* = 0.0263), and cytoplasm by 31.1% (t_(43)_= 3.339, *P* = 0.0017) (Figures 3C–F). The mean intensity difference in IFNγ-exposed processes was not statistically significant (t_(43)_= 1.840, P = 0.0726, unpaired t-test) (Figure 3E). These results show that IFNγ increases MHCI in Day 4 *NGN2*-iNs but has an inverse effect on synapsin I/II, which decreases in the cytoplasm and cell body. Cytoplasmic synapsin I/II and MHCI expression in single cells are positively correlated in the vehicle condition (*r* = 0.57, *n* = 306), which did not change (*P* = 0.1471, z = 1.45) in the IFNγ-exposed condition (*r* = 0.49, *n* = 389).

### Synaptic gene expression is unaltered in IFNγ-exposed neurons

**Figure 4:**
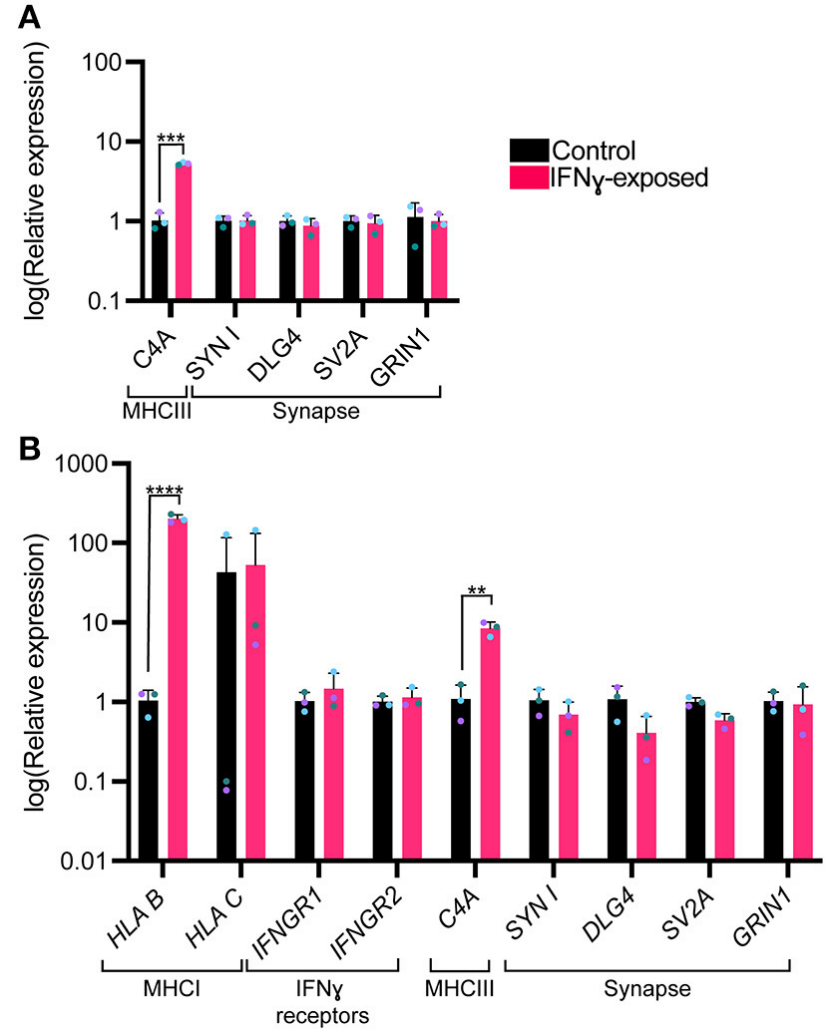
Bar graphs of relative expression of selected synaptic genes and *C4A*, showing increased *C4A* expression in IFNγ-exposed neurons. The bars indicate the log(2^−ΔΔCt^), which indicates the expression relative to housekeepers and normalized to the housekeepers of the control samples (See methods for details.) **(A)** Expression in Day 4 *NGN2*-iNs exposed at day 3. *N* = 3. ****indicates *P* < 0.0001, ***indicates *P* = 0.000291 (unpaired *t*-test). **(B)** Expression in day 27 conventionally differentiated 127_CTM iPSC line NPCs exposed at day 26. ****indicates *P* < 0.0001, **indicates *P* = 0.002947(unpaired *t*-test). The bar represents the mean, the error bars represent the standard deviation. Points of the same color represent the same biological replicate.

We next were interested in understanding whether an acute exposure to IFNγ was sufficient to alter the expression of genes encoding for synaptic genes. Since synapsin I/II decreases in the cell body following IFNγ treatment, we first tested whether expression of synapsin I and other synaptic genes would be decreased following IFNγ treatment. The mean expression level for *SYN1, DLG4, SV2A*, and *GRIN1* were not significantly different from vehicle conditions (Figure 4A). However, when we examined *C4A*, we observed an significant increase in expression of this gene (t_(4)_= 11.84, P = 0.000291). We validated these findings using dual SMAD inhibition differentiated 127_CTM iPSC line NPCs exposed to IFNγ at day 26 to ensure that the observed effects were not cell line specific. As seen in treated NGN2-iNs, IFNγ caused an increase in *HLA-B* (t_(4)_= 22.53, *P* = 0.000023) and *C4A* expression (t_(4)_= 6.466, *P* = 0.002947) in treated NPCs (Figure 4B). No change in *IFNG* receptor expression or of synaptic genes was observed (Figure 4B).

## Discussion

In this study, we used *NGN2*-iNs to study the impact of acute IFNγ exposure on immature developing glutamatergic neurons. We find that IFNγ activates an interferon-mediated canonical signaling pathway in the absence of glial cells and demonstrate that synaptic protein expression is disrupted by this cytokine, building on previous studies showing IFNγ affects expression of synaptic genes in iPSC-NPCs and neurons (28).

The observation that acute IFNγ exposure reduced synapsin I/II expression and increased MHCI expression in immature glutamatergic neurons is consistent with previously published findings. For example, Glynn et al. (31) found an increased density of clusters of synaptic vesicles containing synapsin I upon siRNA knockdown of an MHCI subunit and observed significantly decreased synapsin I at inhibitory synapses when MHCI was overexpressed in rodent neurons. The decrease in synapsin observed in our study is therefore likely linked to the concurrently increased *MHCI* expression, although we observed a positive correlation between synapsin I/II and MHCI intensity at the single-cell level. Decreased synapsin may translate to disruptions in synapses subsequently, as synapsin is important for synapse maturation, including the correct localization of synaptic vesicles in growth cones and the regulation of vesicle recycling rate, although this remains to be tested in *NGN2*-iNs (46). Of note however, a gene enrichment study comparing both rat (gestational day 15, MIA) whole-brain and *post-mortem* human brain tissue samples from individuals with autism reported a common downregulation of genes associated with synaptic vesicle exocytosis (47). This is in line with our SynGO analysis of our IFNy RNAseq datatset (28) and the decrease in synapsin associated with synaptic vesicles observed here. Whether these changes in synaptic protein translate to altered neuronal activity, however, remains to be established. In this context, a previous study suggested that IFNγ treatment of cultured early hippocampal mouse (E15) neurons at 1–4 days *in vitro* had no effect on excitatory transmission, but did not investigate neither other synapse parameters nor whether treatment of NPCs has an effect (24).

Treatment with IFNγ for 24 h did not alter the expression of *selected* synaptic genes. This may be due to several possibilities. For example, changes in proteostasis or mRNA turnover may drive changes in protein levels without affecting mRNA levels. The observed changes in protein levels may reflects a transient change in mRNA expression that is no longer detectable after 24 h. The increase in MHCI gene and protein expression following IFNγ exposure matches our findings in a previous neuroprogenitor cell study that used the same 24-h acute IFNγ exposure of iPSC-NPCs and -neurons (28). This study also described upregulation of *HLA-C* and *HLA-B* expression; however, we only observed a significant increase in *HLA-B*. Consistent with our previous study, we observed no change in the expression of IFNγ receptors. MHCI is known to be involved in synaptic plasticity and learning (29, 30, 48, 49) and is important for negatively regulating synapses (50). Dysregulation of *MHCI* expression could thus potentially be sufficient for a downstream disruption of synapses even if no change in synaptic genes is present at the point of IFNγ exposure. MHCI has been shown to mediate reduced synaptic connectivity in a mouse MIA model by signaling through myocyte enhancer factor 2 (MEF2) (32). Future work would need to establish whether these changes in MHCI expression persist, if MEF2 is involved and whether other downstream changes arise as the neurons mature.

We also observed increased expression of the complement component *C4A* after acute IFNγ exposure. *C4A* mRNA levels are increased in *post-mortem* cortical brain tissue from individuals with schizophrenia and *C4A* variants are associated with elevated risk for schizophrenia (35). The genes downregulated upon increased C4A expression are also enriched for schizophrenia risk (51). C4A is expressed by neurons and colocalizes with synaptic markers and is thought to play a role in the pruning of synapses during brain development and maturation (35). Consistent with this view, overexpression of C4A in mice resulted in behavioral changes of relevance for schizophrenia, reduced cortical synapse density, and increased engulfment of synapses by microglia (52). Inhibition of microglial activity reverses MIA abnormalities, including synapse loss (53). Co-culture studies with microglia are required to understand if IFNγ exposure leads to increased synaptic engulfment by microglia via increased C4A expression. Deletion of C4A could be used to interrogate the role of this protein in the effects of IFNγ in neurons. Co-culture studies with microglia would thus be particularly informative for future IFNγ exposure studies.

We observed activation of the canonical STAT1 signaling pathway following IFNγ exposure but did not observe non-canonical signaling as there was no altered ERK1/2 phosphorylation. This suggests that our observed effects may be mediated by activation of STAT1 signaling, the primary signaling pathway for IFNγ responses (54–56). IFN-γ signaling through JAK/STAT signaling has been observed *in vivo* in multiple species, with effects that promote GABA-ergic inhibition and regulate neuronal connectivity (57). Further experiments are however required to fully interrogate the dynamics of IFNγ signaling pathways in neurons.

In conclusion, elevated levels of IFNγ were sufficient to activate a canonical interferon-signaling mechanism in immature developing neurons, an increase in MHCI proteins and complement components, and reduced synaptic vesicles in immature glutamatergic neurons. Our findings further support a possible link between IFNγ exposure in immature glutamatergic neurons and cellular phenotypes associated with neurodevelopmental disorders, although further work is needed to understand the mechanistic basis of this link.

## Data availability statement

The original contributions presented in the study are included in the article/Supplementary materials, further inquiries can be directed to the corresponding author/s.

## Author contributions

DS and AV: conception and design, literature searching, manuscript writing and editing, project supervision, and financial support. AP, RM, LS, LD, and NA: carried out experiments. AP: manuscript writing and editing. All authors approved the final manuscript.

## Funding

AP, DS, and AV acknowledge financial support for this study from the Medical Research Council (MRC) Centre grant (MR/N026063/1). AP and RM are in receipt of the MRC-Sackler Ph.D. Programme studentship as part of the MRC Centre for Neurodevelopmental Disorders (Medical Research Council MR/P502108/1). LS was supported by the UK Medical Research Council (MR/N013700/1) and King's College London member of the MRC Doctoral Training Partnership in Biomedical Sciences. LD was supported by a research grant from the University of Pennsylvania Autism Spectrum Program of Excellence awarded to DS. DS was also supported by an Independent Researcher Award from the Brain and Behavior Foundation (formally National Alliance for Research on Schizophrenia and Depression (NARSAD) (Grant No. 25957). AV acknowledges financial support for this study from the National Centre for the Replacement, Refinement and Reduction of Animals in Research (NC/S001506/1).

## Conflict of interest

Authors AV and DS receive research funding from bit.bio. The remaining authors declare that the research was conducted in the absence of any commercial or financial relationships that could be construed as a potential conflict of interest.

## Publisher's note

All claims expressed in this article are solely those of the authors and do not necessarily represent those of their affiliated organizations, or those of the publisher, the editors and the reviewers. Any product that may be evaluated in this article, or claim that may be made by its manufacturer, is not guaranteed or endorsed by the publisher.
