# Supplementary material for: Interferon-γ exposure of human iPSC-derived neurons alters major histocompatibility complex I and synapsin protein expression
Source: Front Psychiatry. 2022 Sep 14;13:836217. doi: 10.3389/fpsyt.2022.836217 (PMC9515429; doi:10.3389/fpsyt.2022.836217)
Supplement: Supplementary file 1 [file Data_Sheet_1.PDF]

Supplementary Material

1 Supplementary Figures and Tables

1.1 Supplementary Tables

Supplementary table 1. Media constituents

Medium	Reagent	Manufacturer	Product code	Concentration
N2 medium	DMEM F12	Sigma	D6421	1X
	N2	Life Technologies	17502-048	1%
	Glutamax	Life Technologies	35050-038	2mM
	Non-essential amino acids	Sigma	M7145	0.1%
	2-β Mercaptoethanol	Thermo-Fisher Scientific	21985-023	0.18%
B27 medium	Neurobasal	Life Technologies	21103-049	1X
	B27	Life Technologies	17504-044	2%
	Glutamax	Life Technologies	35050-038	2mM

Supplementary table 2. List of all antibodies used for ICC and western blotting

Antibody name	Dilution	Manufacturer	Product #	Lot #
Mouse anti-HLA-ABC	1:100	Invitrogen	MA5-11723	SF2390832B
Mouse anti-NeuN	1:100	Millipore Sigma	MAB377	2931160
Chicken anti-Nestin	1:500	Novus	NB100-1604	NES88597983
Rabbit anti-Pax6	1:500	BioLegend	901301	B277104
Rabbit anti-β III tubulin	1:500	Abcam	ab18207	GR220660-1
Chicken anti-Synapsin 1/2	1:500	Synaptic Systems	106006	106006/1
Alexa Fluor 488 goat anti-mouse	1:1000	Invitrogen	A11001	2220848
Alexa Fluor 633 goat anti-rabbit	1:1000	Invitrogen	A21071	1932492
Alexa Fluor 568 goat anti-chicken	1:1000	Invitrogen	A11041	1963088
Phospho-Stat1 (Tyr701) rabbit monoclonal antibody	1:1000	Cell Signaling	9167	
GAPDH Mouse McAb	1:20000	Proteintech	60004-I	
Goat anti-mouse IgG Secondary antibody, HRP conjugate	1:10000	Invitrogen	31430	SF252846

Goat anti-rabbit IgG Secondary antibody, HRP conjugate	1:10000	Invitrogen	31460	SH253595

Supplementary table 3. List of primers for qPCR and their sequences

Gene Name	Forward Primer (5'-3')	Reverse Primer (5'-3')
SYN1	CTGGACGTCCCAAACCAC	CTTTCACCTCGTCCTGGCTA
HLA B	TGAGATGGGAGCCGTCTT	ACCTGAACTCTTCCTCCTACA
HLA C	GCTGACCGAGTGAGCCT	TGTTCTTCTTTGATAGCCCATGA
DLG4	GTGACGACCCATCCATTTTC	CTGCCTCTTTGAGGGCTTC
SV-2A	GACGGTGTGGAGGTCTTTGT	CGAAGACGCTGTTGACTGAG
GRIN1	ACTCGGACAAGAGCATCCAC	TCCAGCTGTAGACACGCATC
IFNgR1	GGTCTGTGAAGAGCCGTTGTC	CGGGACCACGTCAGGAATAT
IFNgR2	GGAAAAGGAGCAAGAAGATGTTCT	AGCTCCGATGGCTTGATCTC
C4A	GTTGCTCTTGTTCTCTCCTTCT	CACTGATCCTTTCACTACCTGTC
HPRT	TGACACTGGCAAAACAATGCA	GGTCCTTTTCACCAGCAAGCT
SDHA	AGGAATCAATGCTGCTCTCTGG	CTGCTCCGTCATGTAGTGGA
RPL27	ATCGCCAAGAGATCAAAGATAA	TCTGAAGACATCCTTATTGACG
TBR1	ACACTGGGGCTCACTGGAT	AAAACCACCATCTGCCCATT
PAX6	GCCAGAGCCAGCATGCAGAACA	CCTGCAGAATTCGGGAAATGTCG
MAP2	CTCTCGCACAGAGTTATCC	GACCTACCACCAAGTCCTAAAC
TUBB3	CAAGATGTCCTCCACCTTCATC	GACACCAGGTCGTTCATGTT
RBFOX3	CCTTCCACCGTTTCCTTCTC	GACTTGGTTGGATGCCTCTTATC

Nestin	GAAACAGCCATAGAGGGCAAA	TGGTTTTCCAGAGTCTTCAGTGA

1.2 Supplementary Figures

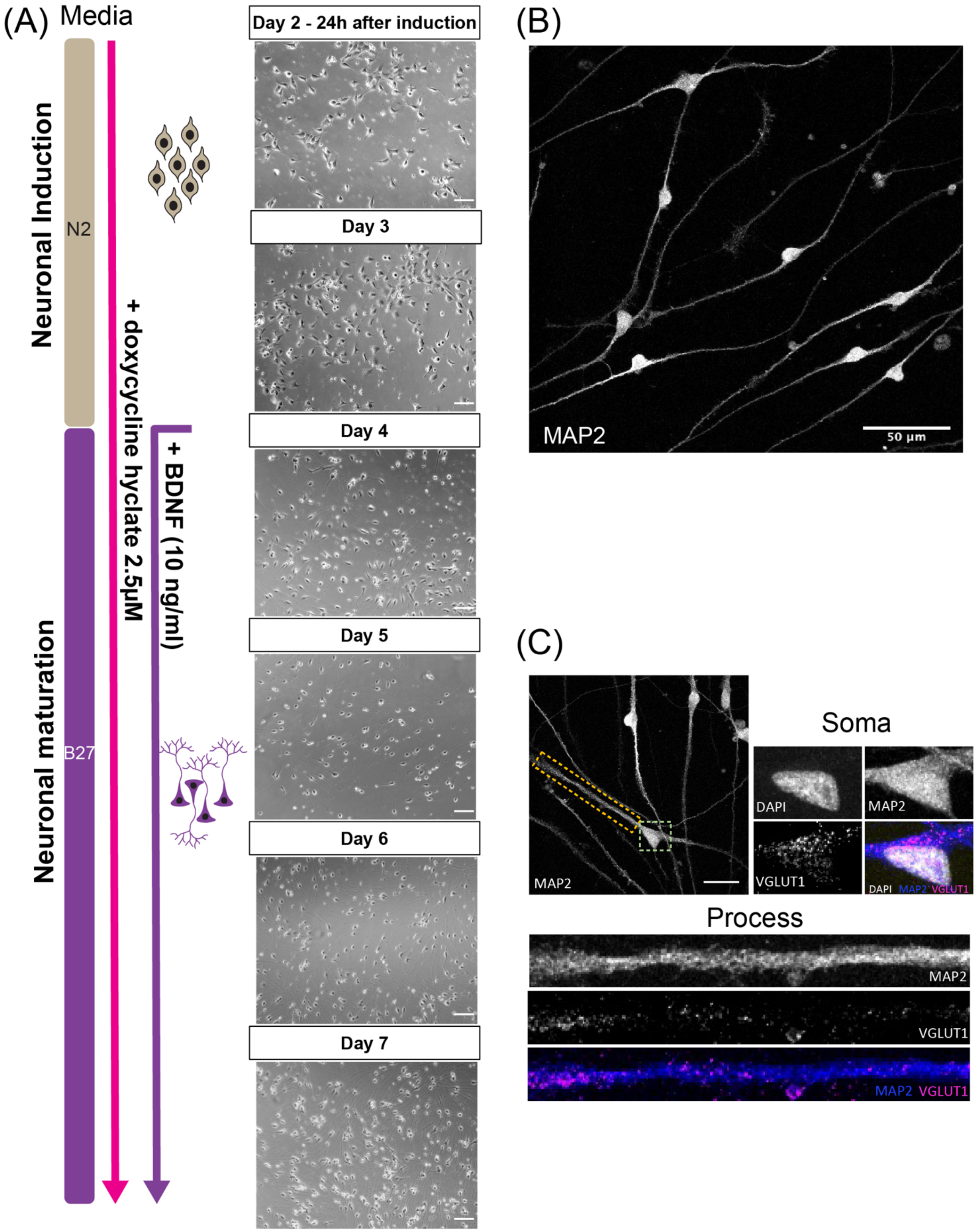

Supplementary Figure 1. NGN2 expression protocol generates glutamatergic neurons**. (A)** Schematic representation and timeline of differentiation protocol. Brightfield images were acquired using an EVOS XL core imaging system with a 10x objective. The scale bars represent 150 µm. **(B)** Representative confocal image of neurons on day 7 after neuronal induction immunostained for neuronal marker microtubule-associated protein 2 (MAP2). Image was acquired using a Leica SP5 confocal microscope with a 63x oil-immersion objective. **(C)** Representative confocal image of neurons on day 7 after neuronal induction immunostained for neuronal marker microtubule-associated protein 2 (MAP2; in blue) and excitatory presynaptic marker vesicular glutamate transporter 1 (VGLUT1; in magenta). The scale bar represents 25 µm. Dotted lines indicate zoomed in regions showing soma and process. At this stage of early neuronal maturation, VGlut1 localises to somatic and process regions. Image was acquired using a Leica SP5 confocal microscope with a 100x oil-immersion objective.

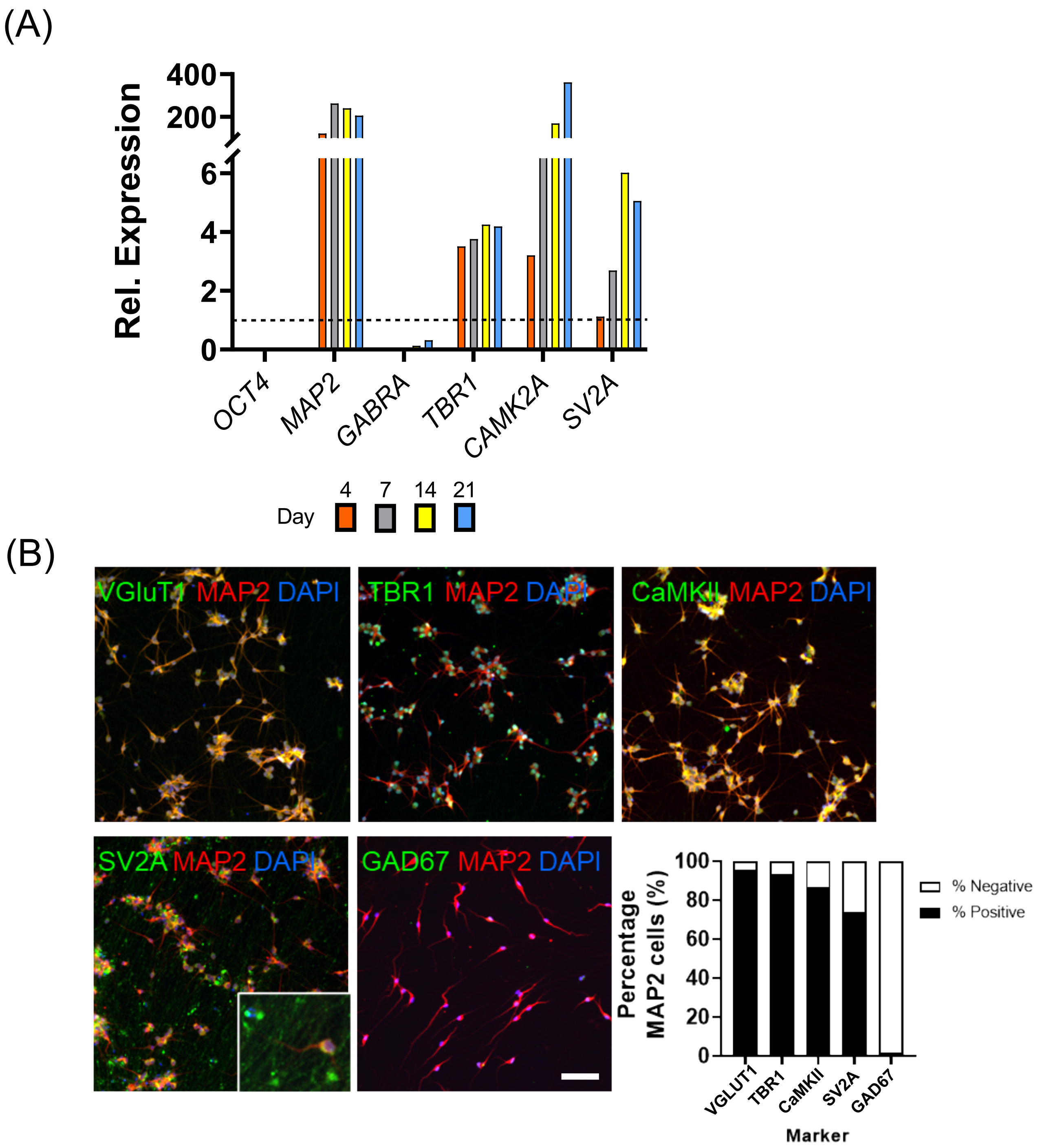

Supplementary Figure 2. Generation of glutamatergic neurons from Opti-OX iPSCs. **(A)** Time-dependent increase in mRNA expression of key fate and synaptic genes indicating the development of excitatory neuronal properties. All expression levels have been normalized to the day 0 time point as a relative expression of 1, indicated by the dotted line. **(B)** After 28 days of differentiation >99% of cells are positive for MAP2 and expressed TBR1, VGLUT1, CaMKII and SV2A consistent with the generation of glutamatergic neurons. In addition, neurons were found to be negative for the

inhibitory neuronal marker, GAD67. Representative images acquired using Thermo Scientific CellInsight High-Content Screening Platform. The scale bar represents 200μm.

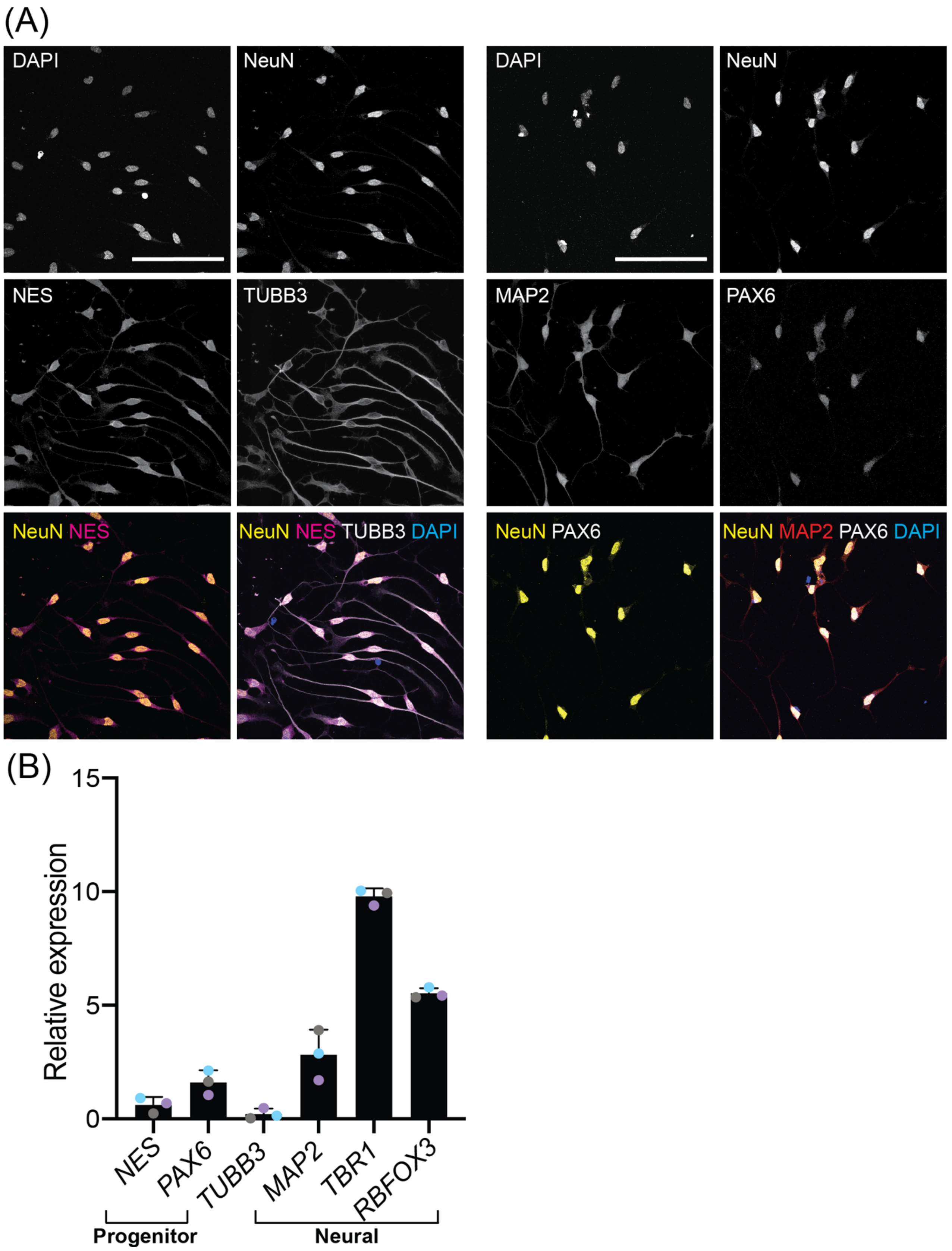
(A)
DAPI
NeuN
DAPI
NeuN
NES
TUBB3
MAP2
PAX6
NeuN NES
NeuN NES TUBB3 DAPI
NeuN PAX6
NeuN MAP2 PAX6 DAPI
(B)
Relative expression
15
10
5
0
NES
PAX6
TUBB3
MAP2
TBR1
RBFOX3
Progenitor
Neural

Supplementary Figure 3. Expression levels of neural and neuroprogenitor genes in D4 Opti-OX cells. **(A)** NeuN, nestin (NES), MAP2, PAX6, and Class III β-Tubulin (TUBB3) expression in day 4 *NGN2*-iNs. Representative images acquired using a Leica SP5 confocal microscope with a 63x oil-immersion objective. The scale bar represents 100µm. **(B)** Bar graph of the relative expression of neural and neuroprogenitor genes, the expression level is the ΔCt value relative to housekeepers. The bar represents the mean, the error bars represent the standard deviation. Points of the same colour represent the same biological replicate.

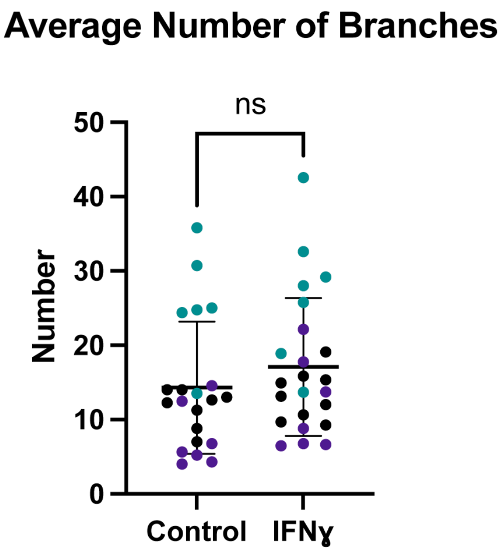
Average Number of Branches
ns
Number
50
40
30
20
10
0
Control
IFNɣ

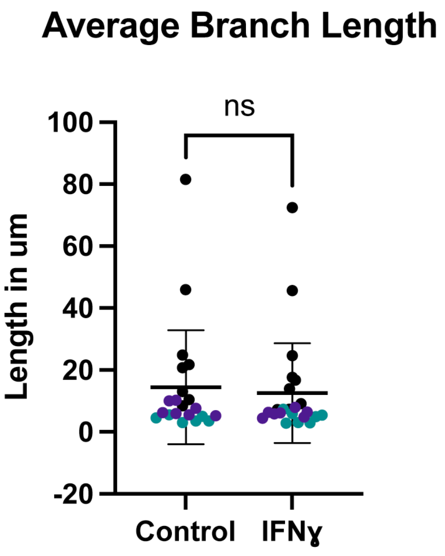
Average Branch Length
ns
Length in um
100
80
60
40
20
0
-20
Control
IFNɣ

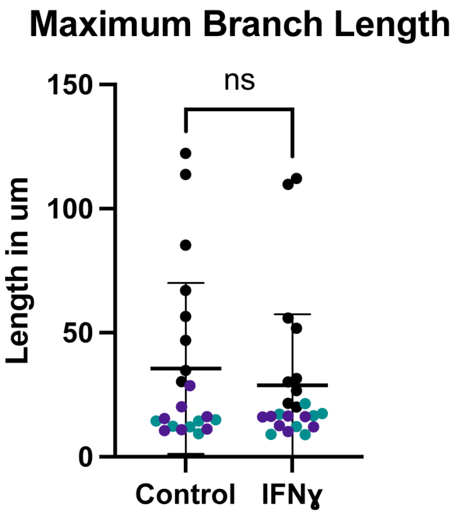
Maximum Branch Length
ns
Length in um
150
100
50
0
Control
IFNɣ

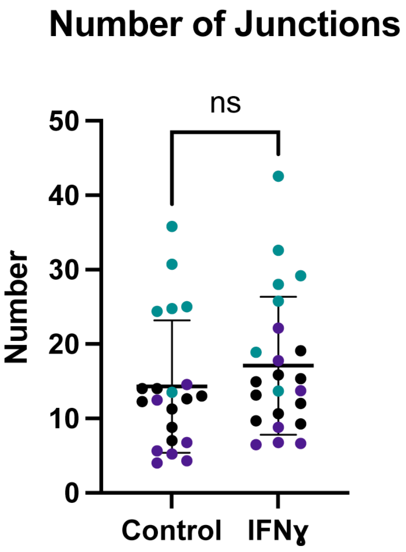
Number of Junctions
ns
Number
50
40
30
20
10
0
Control
IFNɣ

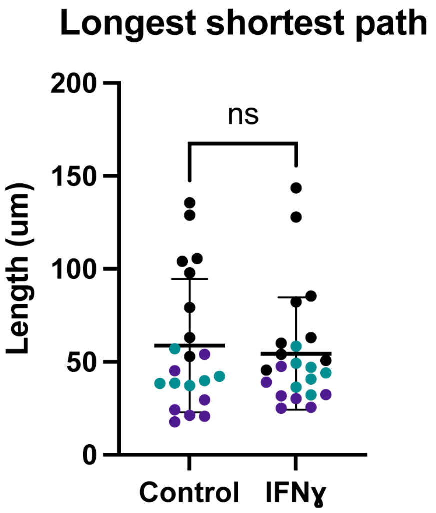
Longest shortest path
ns
Length (um)
200
150
100
50
0
Control
IFNɣ

Supplementary Figure 4. Scatter plots of morphology analysis parameters. Images of neurites were skeletonized using a CellProfiler pipeline and then measured using the Analyze Skeleton (2D/3D) plugin in FIJI. The horizontal bars represent the mean, the error bars represent the standard deviation. Each point in the intensity plots represents the mean intensity of one field of view i.e., image, of the respective object. The different data point colours represent biological replicates with different passage numbers. The IFNɣ and control were compared using an unpaired T-test, where N=3 and ns indicates not significant.
